# Thermal and Fire Behavior of a Bio-Based Epoxy/Silica Hybrid Cured with Methyl Nadic Anhydride

**DOI:** 10.3390/polym12081661

**Published:** 2020-07-26

**Authors:** Aurelio Bifulco, Angela Marotta, Jessica Passaro, Aniello Costantini, Pierfrancesco Cerruti, Gennaro Gentile, Veronica Ambrogi, Giulio Malucelli, Francesco Branda

**Affiliations:** 1 Department of Chemical, Materials and Production Engineering (DICMaPI), University of Naples Federico II, P. le Tecchio 80, 80125 Napoli, Italy; aurelio.bifulco@unina.it (A.B.); angela.marotta@unina.it (A.M.); jessica.passaro@unina.it (J.P.); anicosta@unina.it (A.C.); 2 Institute for Polymers, Composites and Biomaterials (IPCB)-CNR, Via Campi Flegrei 34, 80078 Pozzuoli (NA), Italy; cerruti@ipcb.cnr.it (P.C.); gennaro.gentile@ipcb.cnr.it (G.G.); 3 Department of Applied Science and Technology, Politecnico di Torino, Viale Teresa Michel 5, 15121 Alessandria, Italy; giulio.malucelli@polito.it

**Keywords:** bio-based epoxy resin, silica nanoparticles, in-situ, sol-gel, methyl nadic anhydride, flame retardance

## Abstract

Thermosetting polymers have been widely used in many industrial applications as adhesives, coatings and laminated materials, among others. Recently, bisphenol A (BPA) has been banned as raw material for polymeric products, due to its harmful impact on human health. On the other hand, the use of aromatic amines as curing agents confers excellent thermal, mechanical and flame retardant properties to the final product, although they are toxic and subject to governmental restrictions. In this context, sugar-derived diepoxy monomers and anhydrides represent a sustainable greener alternative to BPA and aromatic amines. Herein, we report an “in-situ” sol–gel synthesis, using as precursors tetraethylorthosilicate (TEOS) and aminopropyl triethoxysilane (APTS) to obtain bio-based epoxy/silica composites; in a first step, the APTS was left to react with 2,5-bis[(oxyran-2-ylmethoxy)methyl]furan (BOMF) or diglycidyl ether of bisphenol A (DGEBA)monomers, and silica particles were generated in the epoxy in a second step; both systems were cured with methyl nadic anhydride (MNA). Morphological investigation of the composites through transmission electron microscopy (TEM) demonstrated that the hybrid strategy allows a very fine distribution of silica nanoparticles (at nanometric level) to be achieved within a hybrid network structure for both the diepoxy monomers. Concerning the fire behavior, as assessed in vertical flame spread tests, the use of anhydride curing agent prevented melt dripping phenomena and provided high char-forming character to the bio-based epoxy systems and their phenyl analog. In addition, forced combustion tests showed that the use of anhydride hardener instead of aliphatic polyamine results in a remarkable decrease of heat release rate. An overall decrease of the smoke parameters, which is highly desirable in a context of greater fire safety was observed in the case of BOMF/MNA system. The experimental results suggest that the effect of silica nanoparticles on fire behavior appears to be related to their dispersion degree.

## 1. Introduction

Thermosetting polymers have been widely used in many industrial applications as adhesives, coatings and laminated materials because of their peculiar physical, chemical, electrical and adhesive properties [1,2]. At present, the reaction of bisphenol A (BPA) and epichlorohydrin, yielding diglycidyl ether of bisphenol A (DGEBA), is the main route for the world production of epoxy prepolymers. Recently, BPA has been banned as a raw material for polymeric products, due to its harmful impact as an endocrine disruptor on human health [3]. In addition, the worldwide environmental issues raised attention regarding the chemical nature of the curing agents. It is well known that for industrial applications of epoxy-based materials, in addition to typical physical and mechanical performance requirements, stringent fire safety regulations must be fulfilled [1]. The use of aromatic amines for the cure of epoxy resins offers good thermal stability together with excellent mechanical and fire performances of the final product, although they are toxic and subject to governmental restrictions [4]. Thus, academic and industrial research is being focused on the design of sustainable green alternatives to BPA and aromatic amines. In this context, considerable research has been carried out on the synthesis of bio-based epoxy resins using compounds derived from vegetable oils, lignin, rosin, tannins, sugar, cardanol or itaconic acid to replace BPA, keeping valuable properties for the final product [5,6,7]. On the other side, the use of anhydride curing agents offers a more sustainable alternative over aromatic and aliphatic amines, even though the products have intermediate mechanical and flame retardant properties [8].

The chemistry of sol-gel may represent a way to design high performing epoxy/silica hybrid nanocomposites. It is well known that it allows silica nanoparticles as small as a few nanometers in diameter be obtained [9,10,11] and is stable because of its large negative ZETA potential. Better epoxy/silica hybrids can be prepared through an in-situ formation of a silicate phase, using sol-gel chemistry. Furthermore, the sol-gel process allows the interface between organic and inorganic phases to be tailored through the adequate selection of both the silane precursors of the inorganic phase and the sol-gel reaction conditions [12,13,14]. The silicate phase may act as a thermal protective layer and reinforcement for the epoxy matrix, improving its mechanical and flame retardant behavior [15,16].

Recently, very few environmentally-friendly and additive-free bio-epoxy resins have been developed [17,18,19]; therefore, the synthesis of renewable sugar-based polymeric materials with mechanical and fire properties similar to their phenyl analogs still represents a great challenge.

2,5-bis[(oxyran-2-ylmethoxy)methyl]furan (BOMF) is one of the most promising furan-based diepoxy monomers for producing sugar-based furan polymers [20,21]. In a recent work, BOMF and DGEBA were cured with methyl nadic anhydride (MNA) in the presence of 2-methyl imidazole (2-MI) as initiator. The study of the thermal behavior of the two epoxy systems showed higher char yields for BOMF epoxy either in nitrogen or air, which could be advantageous in terms of flame retardancy [22]. In similar research, BOMF and DGEBA were cured with diaminodiphenylsulfone (DDS): BOMF-DDS formulations exhibited compact char layers and low heat release rate values, resulting in superior fire resistance over its DGEBA-based counterparts [23].

In this context, using a sol-gel hybrid strategy for the synthesis of silica/BOMF nanocomposites cured with anhydrides may allow green materials to be obtained with satisfactory mechanical and flame retardant properties. In contrast to the phenyl analogs [12,13,15], only few papers have investigated the structure-properties relationships in hybrid furan-based materials [24,25].

Herein, we exploit an in-situ sol–gel silica synthesis procedure for preparing bio-based epoxy/silica nanocomposites, using 2,5-Bis[(oxiran-2-ylmethoxy)methyl]furan (BOMF) diepoxy monomer, cured with methyl nadic anhydride (MNA), in the presence of 2-methyl imidazole (2-MI) as initiator. For comparison, a second set of nanocomposites was prepared, replacing BOMF with a commercial diglycidyl ether of bisphenol A (DGEBA). The morphology, as well as the thermal and flame retardant properties of the hybrid phenyl-based and furan-based silica/epoxy systems were thoroughly investigated by means of infrared spectroscopy (FTIR), transmission electron microscopy (TEM), thermo-gravimetric analysis (TGA) and differential scanning calorimetry (DSC). In addition, UL94 vertical flame spread and forced-combustion (i.e., cone calorimeter) tests were exploited to study the flame retardant behavior of the synthesized systems. The experimental results were compared with those collected from analysis of a traditional phenyl analogue (i.e., DGEBA-based) hybrid resin system cured with aliphatic polyamine (PA). The in-situ approach allowed a very fine distribution of silica nanoparticles (at nanometric level) to be obtained within the epoxy networks, independently from the type of epoxy monomer employed. The hybrid furan-based silica/epoxy systems showed chemical, physical and fire properties comparable to hybrid phenyl-based counterparts, even though these latter showed a remarkably higher glass transition temperature (T_g_).

## 2. Materials and Methods 

### 2.1. Materials

Tetraethyl orthosilicate (TEOS, >99%), (3-aminopropyl)-triethoxysilane (APTES, >98%) and ethanol (ACS reagent, anhydrous) were purchased from Sigma-Aldrich (St. Louis, MO, USA). A commercial epoxy monomer (component A of the resin system SX10) supplied by MATES S.r.l. (Milan, Italy), consisting of a modified bisphenol A diglycidyl ether and a bio-derived furanic diglycidyl ether, namely 2,5-bis[(oxyran-2-ylmethoxy)methyl]furan (BOMF, synthesized on purpose [22]), were used as epoxy monomers. As curing agent for both the epoxy monomers, methyl-5-norbornene-2,3-dicarboxylic anhydride (methyl nadic anhydride, MNA, 90%) purchased from Sigma-Aldrich (St. Louis, MO, USA) was employed. 2-Methylimidazole (2-MI, 99%), purchased from Acros Organics (Fisher Scientific, Hampton, NH, USA), was used as initiator for the curing process.

### 2.2. Preparation of the Epoxy Resins and Epoxy/Silica Hybrid Nanocomposites

Either a fully petroleum-derived or a partially bio-based epoxy system were obtained to respectively cure the commercial epoxy monomer (DGEBA) and the bio-derived one (BOMF), withmethyl nadic anhydride (MNA). For both the epoxides the same protocol was followed: the epoxy monomer was mixed with MNA in order to obtain a homogeneous mixture, and then, the proper amount of initiator was added (0.5 wt. % of 2-MI on the total amount epoxy and anhydride). Once the initiator was well dispersed, the epoxy mixture was poured into a silicon mold, cured for 1 h at 130 °C and post-cured for 1 h at 180 °C in an oven.

A different procedure was adopted for the preparation of epoxy/silica hybrid nanocomposites. More specifically, the coupling agent, 3-Aminopropyl)triethoxysilane (APTS), and the silica precursor, tetraethyl orthosilicate (TEOS), were added to BOMF or DGEBA bis-epoxy monomers, hence promoting an “in situ” sol–gel synthesis prior to the addition of 2-MI and MNA. TEOS loading was kept constant for samples prepared in different batches. The chemical structures of the products used for the production of the epoxy systems and the corresponding hybrids are reported in Table 1.

TEOS/epoxy and TEOS/APTS weight ratios were set at 0.086 and 1.88, respectively. The in-situ formation of the silicate phase required a temperature of 80 °C and reflux conditions. The synthesis procedure was modified and inspired by methodologies reported in the literature [15,22].

The synthesis was performed in one pot involving the following three steps.

1. Mixtures of epoxy (DGEBA) or bio-based epoxy (BOMF) and APTS with epoxy/APTS weight ratio of 23.3 were stirred vigorously at 80 °C for 2 h to get silanized resins.

2. Tetraethoxysilane (TEOS), distilled water and ethanol (EtOH) were added to the silanized resins and stirred vigorously at 80 °C under reflux for 90 min. Subsequently, the reaction vessels were opened and kept at 80 °C for 30 min in order to remove ethanol and water.

3. The amounts of 2-MI and MNA needed for the curing were then added to the mixtures at room temperature. The resulting mixtures were degassed under vacuum and poured into a silicon mold. For each formulation the thermoset networks were obtained by applying the following thermal treatment: cure at *T* = 130 °C for *t* = 1 h; post-cure at *T* = 180 °C for *t* = 1 h.

The silica content estimated from the stoichiometry was 2 wt. %. The typical reaction batches are presented in Table 2 together with their acronyms, which will be used throughout the paper. DGEBA/MNA indicates the pristine epoxy system based on DGEBA, while BOMF/MNA represents the bio-epoxy system derived from BOMF. “2Si” in the acronym means that the epoxy network contains 2 wt. % of in-situ silica nanoparticles.

### 2.3. Methods

A PerkinElmer Spectrum One FTIR spectrophotometer (PerkinElmer, Norwalk, CT, USA) equipped with a universal ATR sampling accessory and Spectrum Software (v10.5.1, PerkinElmer, Wellesley, MA, USA) was used to perform FT-IR analysis in ATR mode. Spectra were acquired in the range of 4000–600 cm^−1^ with a resolution of 4 cm^−1^ and 32 scans. 

Thermogravimetric analysis (TGA) was performed by means of a TA Instrument TGA 500 (TA-Instruments, New Castle, DE, USA) and Universal Analysis 2000 Software (v4.5.0.5, New Castle, DE, USA), heating each sample from 25 to 750 °C at 10 °C/min, under nitrogen and air with a flow of 60 mL/min.

Differential scanning calorimetry (DSC) was carried out by means of a TA Instrument DSC Q2000 (TA-Instruments, New Castle, DE, USA) and Universal Analysis 2000 Software (v4.5.0.5, New Castle, DE, USA), applying the following protocol: the sample was equilibrated at −60 °C, than heated up to 200 °C at 10 °C/min, cooled down to −60 °C at the same rate, kept at that temperature for 1 min and heated up again to 250 °C. All the analyses were performed under a 30 mL∙min^−1^ nitrogen flux. The glass transition temperature (*T*_g_) was calculated as the midpoint between onset and endpoint of the specific heat variation.

Transmission electron microscopy (TEM) was performed on a FEI TECNAI G12 Spirit-Twin operating at 120 kV (Hillsboro, OH, USA) and LaB6 source. The microscope was equipped with a FEI Eagle 4K CCD camera and NanoImaging Services Software (v4, San Diego, CA, USA).

UL94 flame spread tests were performed according to the D3801−10 standard; the size of the specimens was 120 × 10 × 2 mm^3^. 

Cone calorimetry tests (Fire Testing Technology, East Grinstead, London, UK) were performed according to the ISO 5660 standard, using squared samples (10.0 × 10.0 × 0.3 cm^3^), with a heat flux of 35 kW/m^2^, in horizontal configuration. Time to ignition (TTI, s), total heat release (THR, MJ/m^2^), peak of the heat release rate (pkHRR, kW/m^2^) and heat release rate (HRR, kW/m^2^) were measured. Total smoke release (TSR, m^2^/m^2^), carbon monoxide yield (CO yield, kg/kg), carbon dioxide yield (CO_2_ yield, kg/kg) and specific extinction area (SEA, m^2^/kg) were evaluated as well. For each sample, the experiments were repeated at least three times in order to ensure reproducible and significant data.

## 3. Results and Discussion

### 3.1. Characterization of the Epoxy/Silica Hybrid Nanocomposites

The effective formation of silica nanoparticles was monitored using infrared analysis.

**Figure 1:**
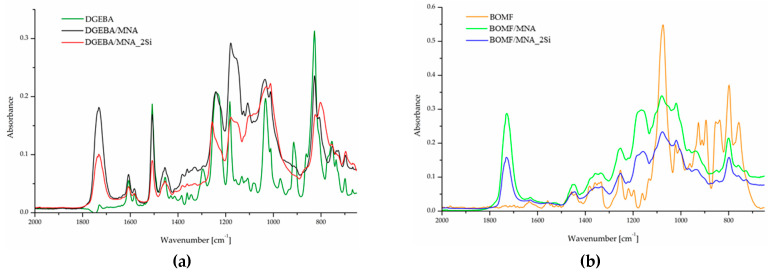
FTIR-ATR spectra of (**a**) DGEBA, the DGEBA-based epoxy resin (DGEBA/MNA) and epoxy/silica hybrid (DGEBA/MNA_2Si), and (**b**) BOMF, the furan-based epoxy resin (BOMF/MNA) and epoxy/silica hybrid (BOMF/MNA_2Si).

Figure 1a,b shows the FTIR-ATR spectra of the epoxy resins, before and after curing and of the corresponding hybrid nanocomposites.

**Figure 2:**
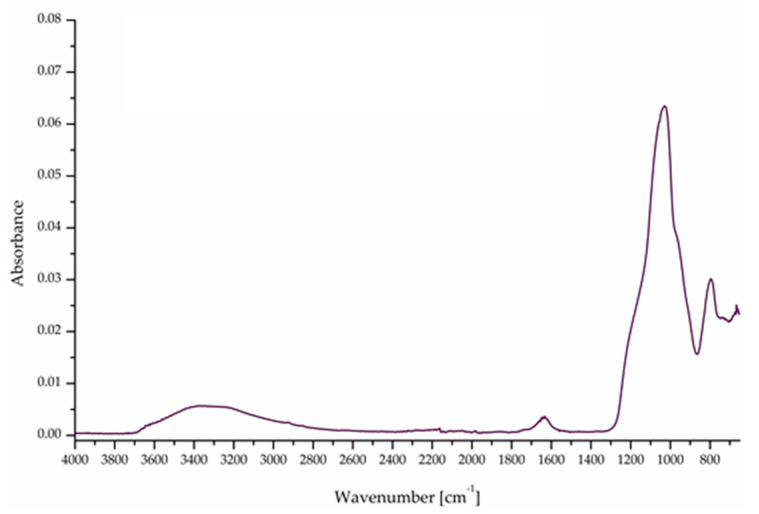
FTIR-ATR spectra of the thermogravimetric analysis (TGA) residue of BOMF/MNA_2Si in air.

The formation of the ester bond, characteristic of the reaction between the anhydride and oxirane group, is confirmed in all the samples by the appearance of strong peaks at about 1730 cm^−1^. The presence of silica is also noticeable in the DGEBA/MNA_2Si sample (Figure 1a), where a strong peak at 1010 cm^−1^appears. Conversely, no clear evidence of silica formation is detectable in the FTIR-ATR spectra of BOMF/MNA_2Si (Figure 1b), as the Si–O–Si signal superimposes with other peaks; the effective formation of silica in the epoxy structure is anyway confirmed using the FTIR-ATR spectra performed on the residue collected after TGA analyses in air (Figure 2), where the well-known SiO_4_ stretching vibration band is clearly recognizable.

### 3.2. Morphology and Structure of the Obtained Hybrid Systems

**Figure 3:**
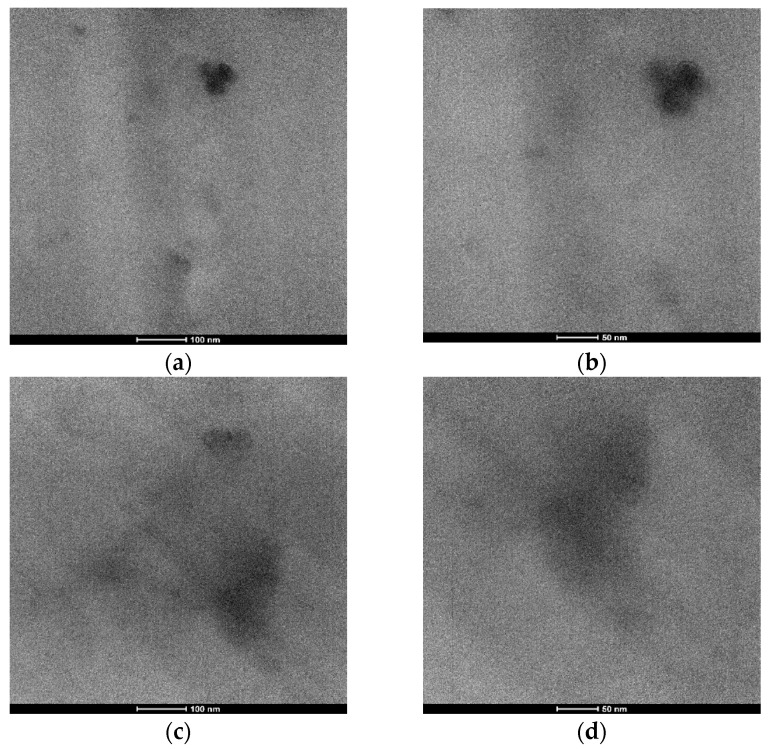
Typical TEM micrographs for (**a**,**b**) BOMF/MNA_2Si and (**c**,**d**) DGEBA/MNA_2Si at different magnifications.

The typical TEM micrographs for BOMF/MNA_2Si and DGEBA/MNA_2Si are shown in Figure 3a,b and Figure 3c,d, respectively. For both the epoxy systems, a very fine distribution of silica nanoparticles embedded in a hybrid co-continuous network can be observed. In addition, as far as BOMF/MNA_2Si is considered, some particles tend to aggregate into clusters or bigger particles (see Figure 3a,b). Due to the significantly lower viscosity of BOMF, compared to DGEBA, indeed, a more significant molecular motion is allowed, leading to the formation bigger silica clusters.

It’s worth underlining that the synthesis strategy of the present work, described in the experimental section, is similar to the one adopted in a previous paper [15] but differs for the final curing step (i.e., step 3, for which DGEBA was cured with polyamines for 4 h at 80 °C). However, in any case, performing the sol-gel method allows nanocomposites with a nanometric distribution of silica nanoparticles to be obtained.

### 3.3. Thermal Analysis

**Figure 4:**
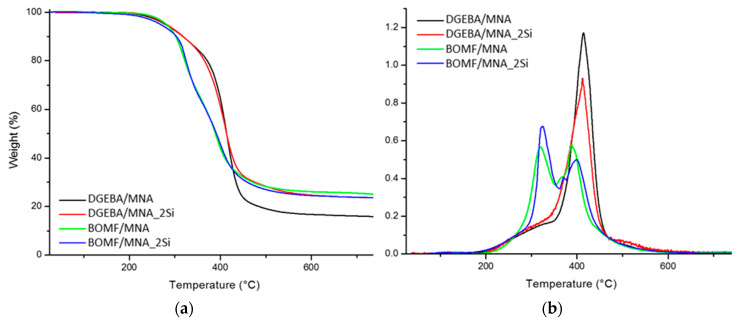
(**a**) TGA and (**b**) derivative curves (DTG) thermograms of epoxy resins and epoxy/silica hybrids.

Figure 4 shows the thermogravimetric (TGA) and derivative curves (DTG) in inert atmosphere for pristine epoxy resin systems (i.e., DGEBA/MNA and BOMF/MNA) and the corresponding hybrids (i.e., DGEBA/MNA_2Si and BOMF/MNA_2Si). Table 3 shows the data collected using TGA analysis: *T*_5_, *T*_10_ and *T*_50_ are the temperatures at which 5%, 10% and 50% weight loss are recorded; the residues at 700 °C (R_700_) are also reported.

In nitrogen, any epoxy system degrades according to a two-step mechanism [26,27]. The first degradation, which occurs around 280 °C, is ascribed to the release of some principal volatile products, specifically acrolein, acetone and allyl alcohol. The second degradation refers to the formation of high molecular weight products along with more complex phenolic compounds, which evolve during the main decomposition step of the cross-linked resin (beyond 340 °C) [27]. In inert atmosphere, no weight changes are generally observed between 400–600 °C, because of the formation of a very stable aromatic char.

It is worthy of note that the presence of silica nanoparticles does not significantly modify the degradation mechanism of DGEBA/MNA and BOMF/MNA, which substantially follows the typical decomposition path described above, notwithstanding a slight decrease of *T*_5_ for BOMF/MNA_2Si with respect to the other systems. However, the degradation of both BOMF/MNA and BOMF/MNA_2Si samples occurs with higher pyrolysis rates with respect to DGEBA-based systems, as confirmed by the TG and DTG curves and *T*_10_ and *T*_50_ values. It is also worth noting the very high residues at 700 °C (about 24%), obtained for all the systems but DGEBA/MNA (for which the residue is lower, though about 16%).

Table 3 also collects the glass transition temperature (*T*_g_) values. The presence of the in situ-formed silica increases the *T*_g_ of the DGEBA/MNA system. Conversely, the hybrid strategy seems to negatively influence the BOMF-based system, for which a decrease of *T*_g_ was recorded in the corresponding hybrid. These results suggest that strong interactions occur at the silica/matrix interface in the case of the DGEBA/MNA system, for which silica clustering, as assessed using TEM analyses, is negligible. At variance, the formation of aggregates in the BOMF/MNA system lowers the interactions between the two phases in the hybrid system, hence lowering the *T*_g_.

### 3.4. Flame Retardant Behavior

The investigation of the overall fire behavior of a flame retarded system is usually carried out by performing either flame spread or forced combustion (i.e., cone calorimetry) tests.

The flammability of all the studied systems was tested through UL94 vertical spread test. All the samples (i.e., DGEBA/MNA, DGEBA/MNA_2Si, BOMF/MNA and BOMF/MNA_2Si) resulted as being not classifiable according to the UL94 vertical spread test, because it was not possible to prevent the flame propagation along the sample, as already observed for DGEBA cured with polyamines (PAs) [15]. However, a strong influence of the use of the anhydride hardener on the combustion behavior and, in the case of the DGEBA/MNA system, of silica addition was observed.

**Figure 5:**
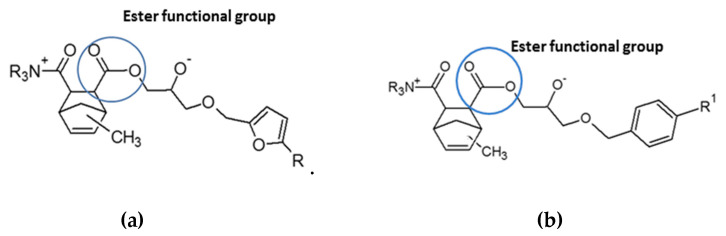
The curing of BOMF (**a**) and DGEBA (**b**) monomers leads to the formation of an ester group between the MNA and epoxy groups.

It is worth pointing out that, in vertical flame spread tests, the DGEBA/MNA and BOMF/MNA systems do not exhibit dripping phenomena when exposed to a Bunsen flame, even in the absence of silica [28]. Conversely, dripping occurred when DGEBA was cured with polyamines [15]. This peculiar behavior may be ascribed to the use of an anhydride as curing agent. More specifically, the propagation process that occurs during the curing of DGEBA and BOMF leads to the formation of an ester group between MNA and the epoxy group, as confirmed using FTIR results (see Figure 1 and Figure 5). The heat that is provided during the combustion process may decompose the ester group, generating an acid adduct of the methyl nadic anhydride, together with secondary products [29,30,31]. The charring ability of epoxy resins is well known; this is due to their degradation path that occurs mainly through a carbonization process (i.e., dehydration reactions) [32]. The production of acid species from the thermal decomposition of ester functional groups in the final products may promote the carbonization process, through dehydration reactions occurring between acid compounds and the epoxy resin [2,33,34,35]. The use of an anhydride curing agent may strongly contribute to boost the char formation in the boundary layer (i.e., charring region), with respect to an aliphatic amino system [15]. The MNA effect on the char-forming behavior of the epoxy polymer may affect the melt viscosity of the burning system, hence inhibiting the formation of incandescent drops during the combustion. 

**Figure 6:**
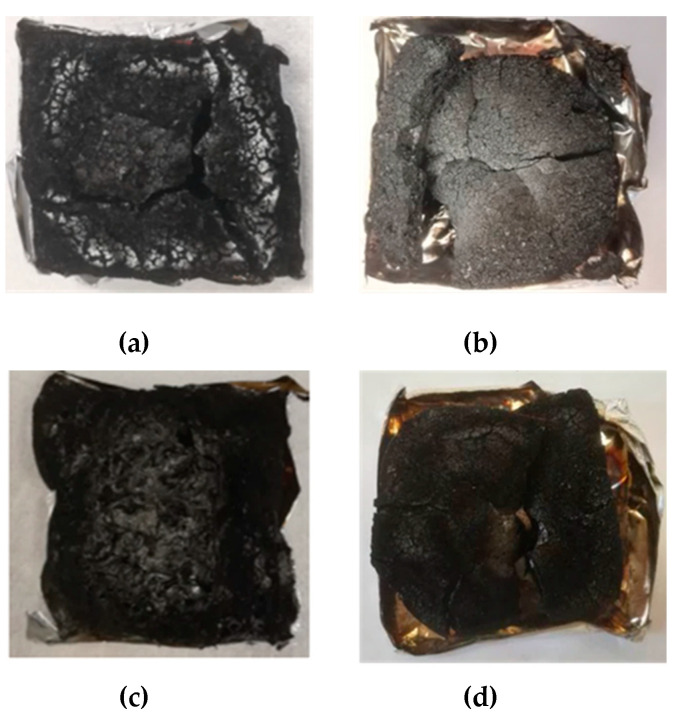
Photographs of the char residues obtained after cone calorimetry tests for (**a**) DGEBA/MNA, (**b**) BOMF/MNA, (**c**)DGEBA/MNA_2Si and (**d**) BOMF/MNA_2Si.

As far as forced-combustion tests are considered, Table 4 collects the thermal parameters in terms of TTI, HRR, pkHRR, THR and final residue for all the samples investigated. The cone calorimetry results for the system DGEBA/PA (i.e., DGEBA/Polyamine [15]) are also reported for comparison. The residues at the end of the tests show that around 10% of the initial mass of samples cured with MNA is preserved for all the formulations, which, together with the pictures of Figure 6, further demonstrates the high char-forming character of the epoxy systems cured with MNA. This is in very good agreement with the high residues of TGA analyses (see Section 3.3) and with the explanation given above for the absence of dripping phenomena. In addition, a strong reduction of HRR of DGEBA/MNA (−25%) and BOMF/MNA (−35%) was recorded with respect to the DGEBA/PA system (Table 4). This result, which seems to be mainly ascribed to the use of MNA as hardener, can be further explained by the higher char formation in the boundary layer (i.e., charring region), induced by the acidic compounds produced during the thermal degradation of the MNA-cured samples.

Interestingly, a further strong reduction (−40%) of HRR was recorded as the effect of the addition of silica to the DGEBA/MNA system; this value is very close to that recorded (38%) for the DGEBA/PA system containing silica. When comparing HRR of DGEBA/MNA containing 2 wt. % of silica with the corresponding system cured with PA, HRR is lowered by 55% as the result of effects provided by MNA and silica. Conversely, no remarkable decrease of HRR was observed upon addition of silica to BOMF/MNA. This finding is in agreement with results [36] obtained for PMMA (polymethyl methacrylate) incorporating carbon nanotubes. For this system, it was found that the HRR values of the sample with a good nanotube dispersion are much lower than those of unfilled PMMA and of the sample showing poor dispersion. Accordingly, in the present case the HRR does not change significatively upon addition of silica in the case of the BOMF/MNA system, where the dispersion of silica is not adequate.

As reported in the literature [2,15,37,38], hybrid systems are expected to anticipate the ignition (TTI) as compared to the neat cured resins. This anticipation does not occur in the case of DGEBA/MNA_2Si and is almost negligible in the case of BOMF/MNA_2Si.

Based on the results discussed so far, BOMF cured with MNA allows the prevention of melt dripping phenomena, showing a char-forming character comparable to the DGEBA-based system. At the same time, the flame retardant properties for the bio-epoxy resin (i.e., BOMF/MNA) appear unchanged with respect to the phenyl analog. However, MNA and silica nanoparticles mainly act in the condensed phase activity, as revealed by CO/CO_2_ ratio values (Table 5), which are almost unchanged with respect to the neat epoxy systems. More specifically, the combination of MNA and silica leads to the formation of a stable char working as a thermal shield and oxygen barrier (Figure 6) [39]. 

Finally, as far as the smoke parameters (see Table 5) are concerned, the bio-epoxy resin and its hybrid system show an overall decrease of TSR, SEA, CO and CO_2_ yields, compared to DGEBA/PA, DGEBA/PA_2Si, DGEBA/MNA and DGEBA/MNA_2Si. It is well known that soot is one of the major decomposition products during the degradation of an epoxy resin, because of its tendency to produce char and smoke. It is reported in the literature that soot includes species with high molecular weight formed in the gas-phase combustion process and residual pyrolyzed fuel particles [40]. During the last steps of the carbonization and gas-phase combustion processes of a DGEBA resin, several phenyl-based compounds (e.g., benzene, naphthalene, anthracene, among a few to mention) are produced at high temperatures (800–1000 °C) [41]. Furan-based epoxy polymers are reported to produce a lower amount of high molecular weight compounds, despite an increased residual char, because of the furan structure that reduces the possible cyclization reactions [23,42,43]. Therefore, the combustion of BOMF/MNA and BOMF/MNA_2Si leads to a lower number of products constituting the black soot composition, with respect to DGEBA/MNA and DGEBA/MNA_2Si counterparts.

## 4. Conclusions

In the present work, an in-situ sol-gel synthesis was performed on a furan-based diepoxy monomer, namely 2,5-bis[(oxyran-2-ylmethoxy)methyl]furan (BOMF) and the obtained epoxy/silica hybrid nanocomposite was thoroughly investigated and compared to the hybrid system prepared using diglycidyl ether of bisphenol A (DGEBA). Methyl nadic anhydride (MNA) was used as curing agent, in the presence of 2-methyl imidazole (2-MI), in order to use a green approach as much as possible for the synthesis processes. Both systems were compared with the hybrid one prepared using diglycidyl ether of bisphenol A (DGEBA) cured with apolyamine.

Morphological study of the furan-based epoxy/silica nanocomposite showed that promoting the formation of silica through the sol-gel route (step 2) in the presence of a pre-formed hybrid epoxy/APTS adduct (i.e., during step 1), in different systems, allowed nanocomposites to be obtained with a very fine distribution of silica nanoparticles (size of a few nanometers). Some tendency of the particles to aggregate into clusters or bigger particles was observed in the case of the BOMF/MNA system only.

The thermal stability of both epoxy systems was not significantly affected by the presence of silica nanoparticles, although the BOMF-based samples (both unfilled and hybrid) showed higher pyrolysis rates and residues with respect to DGEBA-based systems. In agreement with the morphological study, an increase of the glass transition temperature was observed for DGEBA-based epoxy/silica nanocomposites, for which a homogenous and very fine distribution of silica was observed. 

As concerns the fire behavior, BOMF and DGEBA monomers cured with anhydride did not suffer melt dripping phenomena and showed high char-forming character and thus a remarkable decrease (−35% and −25%, respectively) of the heat release rate compared to the DGEBA-based systems cured with an aliphatic polyamine. In addition, the bio-epoxy resin and its hybrid system showed an overall decrease of the smoke parameters compared to their DGEBA-based counterparts.

These results demonstrate that BOMF-based resins cured with an anhydride may represent a suitable green alternative to DGEBA-based systems in the synthesis of epoxy/silica hybrid nanocomposites, as the furan-based epoxy/silica nanocomposite showed morphological characteristics and thermal properties similar to the “bisphenol A”-based counterpart. In addition, the combined use of BOMF monomer with anhydride as curing agent was able to lower smoke production during forced-combustion tests. Finally, the experimental results suggest that the dispersion degree of silica within the designed epoxy networks plays a key role in determining their overall final properties.

## Figures and Tables

**Table 1:** Chemical structure and related nomenclature of the products used for the synthesis of epoxy systems and of the corresponding hybrids.

Molecule Structure	Nomenclature
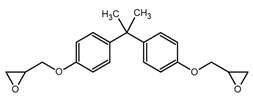	Diglycidyl ether of bisphenol A (DGEBA)
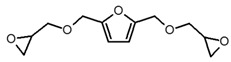	2,5-Bis[(oxiran-2-ylmethoxy)methyl]furan (BOMF)
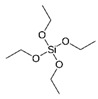	Tetraethyl orthosilicate (TEOS)
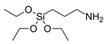	3-Aminopropyl)triethoxysilane (APTS)
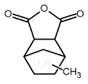	Methyl nadic anhydride (MNA)
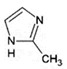	2-methyl imidazole (2-MI)

**Table 2:** Acronyms and batch compositions of the epoxy and epoxy/silica hybrids studied.

Sample	DGEBA (g)	BOMF (g)	TEOS (g)	APTS (g)	EtOH (g)	H_2_O (g)	2-MI (g)	MNA (g)
DGEBA/MNA	43.8	---	---	---	---	---	0.32	20.7
DGEBA/MNA_2Si	45.0	---	3.89	2.00	0.50	1.73	0.31	21.3
BOMF/MNA	---	36.8	---	---	---	---	0.32	27.3
BOMF/MNA_2Si	---	45.0	3.89	2.00	0.50	1.73	0.39	32.6

**Table 3:** Values of glass transition temperature (*T*_g_) measured using differential scanning calorimetry (DSC), temperatures at which 5% (*T*_5_), 10% (*T*_10_) and 50% (*T*_50_) weight loss are recorded and residues weight at 700 °C (R_700_), collected using TGA analysis.

Sample	T_g_ (°C)	T_5_ (°C)	T_10_ (°C)	T_50_ (°C)	R_700_ (wt. %)
DGEBA/MNA	65	279	317	414	16
DGEBA/MNA_2Si	72	278	316	410	24
BOMF/MNA	53	281	302	389	26
BOMF/MNA_2Si	43	268	303	391	24

**Table 4:** Results from cone calorimetry tests performed on epoxy resins and epoxy/silica hybrids. TTI, time to ignition; HRR, heat release rate; pkHRR, peak of the heat release rate; THR, total heat released.

Sample.	TTI (s)	HRR (kW/m^2^)	pkHRR (kW/m^2^)	THR (MJ/m^2^)	Residue Mass (%)
DGEBA/PA *	54 ± 3	504 ± 23	1971 ± 384	84 ± 3	2 ± 0.7
DGEBA/PA_2Si *	37 ± 4	311 ± 12	991 ± 73	67 ± 9	6 ± 0.5
DGEBA/MNA	43 ± 3	377 ± 9	1119 ± 39	94 ± 6	8 ± 1
DGEBA/MNA_2Si	54 ± 3	227 ± 9	625 ± 33	96 ± 1	11 ± 1
BOMF/MNA	49 ± 3	333 ± 4	1216 ± 37	75 ± 6	10 ± 1
BOMF/MNA_2Si	45 ± 2	327 ± 4	983 ± 11	76 ± 2	11 ± 1

* DGEBA/PA and DGEBA/PA_2Si represent a DGEBA-based epoxy system cured with polyamine agent and epoxy/silica hybrid sample, respectively, which were studied by the authors in a previous paper [15].

**Table 5:** Smoke results from cone calorimetry tests performed on epoxy resins and epoxy/silica hybrids. TSR, total smoke release; SEA, specific extinction area.

Sample	TSR (m^2^/m^2^)	SEA (m^2^/kg)	CO Yield (kg/kg)	CO_2_ Yield (kg/kg)	CO/CO_2_ratio
DGEBA/PA *	3066 ± 206	940 ± 36	0.06 ± 0.03	2.1 ± 0.06	0.028
DGEBA/PA_2Si *	2604 ± 291	941 ± 38	0.06 ± 0.04	1.9 ± 0.03	0.031
DGEBA/MNA	3619 ± 292	835 ± 8	0.05 ± 0.01	1.9 ± 0.1	0.026
DGEBA/MNA_2Si	3864 ± 309	808 ± 5	0.06 ± 0.01	1.8 ± 0.2	0.033
BOMF/MNA	2086 ± 242	479 ± 9	0.05 ± 0.01	1.8 ± 0.1	0.027
BOMF/MNA_2Si	1802 ± 147	444 ± 8	0.05 ± 0.01	1.9 ± 0.1	0.026

* DGEBA/PA and DGEBA/PA_2Si represent a DGEBA-based epoxy system cured with a polyamine with and without in-situ generated silica, respectively, which were studied by the authors in a previous paper [15].
